# Compressive Strength of Mineral Trioxide Aggregate and Calcium-enriched Mixture Cement Mixed with Propylene Glycol

**DOI:** 10.22037/iej.v12i4.17748

**Published:** 2017

**Authors:** Fereshte Sobhnamayan, Alireza Adl, Nooshin Sadat Shojaee, Mahdi Sedigh-Shams, Elnaz Zarghami

**Affiliations:** a *Department of Endodontics, Dental School, Shiraz University of Medical Sciences, Shiraz, Iran; *; b *Department of Endodontics, Biomaterials Research Center, Dental School, Shiraz University of Medical Sciences, Shiraz, Iran; *; c *Student Research Committee, Dental School, Shiraz University of Medical Sciences, Shiraz, Iran *

**Keywords:** Calcium-Enriched Mixture Cement, Compressive Strength, Mineral Trioxide Aggregate, Propylene Glycol

## Abstract

**Introduction:**

The aim of the present study was to evaluate and compare the compressive strength (CS) of mineral trioxide aggregate (MTA) and calcium-enriched mixture (CEM) cement when mixed with propylene glycol (PG).

**Methods and Materials:**

Twenty four custom-made split molds with 5 holes in each were prepared. Molds were allocated into eight groups (*n*=15 holes) as follows: Groups 1,5: CEM and MTA mixed with PG (100%), Groups 2,6: CEM and MTA mixed with PG (20% )+CEM or MTA liquid (80%) respectively, Groups 3,7: CEM and MTA mixed with PG (50% )+CEM or MTA liquid (50% ) respectively, Groups 4,8: CEM and MTA mixed with CEM or MTA liquid respectively as control groups. All specimens were kept in 37^°^C in an incubator and the compressive strength was evaluated after 7 days. Data were analyzed using the Kruskal Wallis and Dunne tests. The level of significance was set at 0.05.

**Results:**

In all concentration of PG, MTA samples showed better results than CEM cement. In CEM samples, adding 20% PG could significantly increase the compressive strength in comparison with control group and 100% PG (*P*=0.047 and *P*=0.011, respectively). In MTA samples, adding 100% and 50% PG significantly increased the compressive strength of the cement in comparison with control group (*P*=0.037 and, *P*=0.005, respectively).

**Conclusion:**

Considering the limitations of the present study, appropriate concentration of PG could improve the CS of MTA and CEM cement.

## Introduction

Mineral trioxide aggregate (MTA) is a hydrophilic calcium silicate-based cement with osteogenic, cementogenic and odontogenic potential that can be used for perforation repairs, pulp capping and pulpotomy [1, 2].

Distilled water is normally used for mixing MTA. However this mixture is difficult to manipulate and its setting time is long [3, 4].

In order to alleviate these problems other vehicles have been proposed but, the clinical effects are controversial. Methylcellulose, calcium chloride, calcium lactate gluconate, PG and KY liquid (Johnson & Johnson, Langhorne, PA, USA) are among the vehicles that improve the manageability of this mixture [3, 5-7]. PG is a nontoxic alcoholic viscose vehicle that successfully improved the handling of MTA [6, 8, 9]. It also increase its push-out bond strength [10], sealing ability [8] and results in higher pH and Ca^2+^ dissociation during the initial post-mixing periods [6, 9].

Different ratios of PG and water has been shown to affect the physical and chemical properties of this cement, as crystal hydration is an important factor for the setting reaction of MTA [11]. The addition of high ratios of PG (≥50%) decreases the water content in the mixture and causes the changes in physical and chemical characteristics of MTA [9].

Compressive strength (CS) is an indicator of setting reaction and stability of the materials [12, 13]. This entity in hydraulic cements such as MTA is as an indicator of hydration reaction which is affected by the type of MTA, the mixing liquid, condensing pressure and the techniques used for mixing the powder and liquid [14-17].

Calcium-enriched mixture (CEM) cement is another hydrophilic cement with clinical applications similar to MTA but a different chemical composition [18-21]. This novel endodontic cement showed favorable results in terms of biocompatibility, antibacterial effect and sealing properties [19, 22-26]. Different studies showed that different mixing methods and vehicles could affect the compressive strength of this material, as well [16, 27]. So far, there have been no published studies on the effect of PG on the CS of CEM cement. Therefore, this *in vitro* study aimed to evaluate the effect of adding different ratios of PG into MTA liquid and CEM liquid on the CS of these materials during seven days post mixing.

## Materials and Methods

Twenty four custom-made two-part split Plexiglass molds were used in this experimental *in vitro* study. Each mold had five holes with internal diameter of 4±0.1 mm and height of 6±0.1 mm. The molds were randomly allocated into eight groups (3 molds/15 holes in each group). The groups comprised groups 1 and 5; CEM and MTA mixed with PG (100%), groups 2 and 6; CEM and MTA mixed with PG (20%)+CEM or MTA liquid (80%), respectively, groups 3 and 7; CEM and MTA mixed with PG (50%)+CEM or MTA liquid (50%), respectively, and groups 4 and 8; CEM and MTA mixed with CEM or MTA liquid, respectively as control groups (Table 1). The CEM or MTA liquid /PG ratios were determined by volume. The powder/liquid ratio was 1 g powder to 0.4 mL liquid for MTA based on a previous study and 1 g powder to 0.54 mL liquid for CEM cement based on a pilot study.

CEM (BioniqueDent, Tehran, Iran) and MTA (Angelus; Londrina, Parana, Brazil) were prepared as above and then homogenized and immediately positioned incrementally into the

molds by amalgam carrier. After gentle packing and compacting with condensers, excess material was removed with wet cotton pellets. The molds were then wrapped into wet pieces of gauze saturated with PBS and kept in an incubator at 37^°^C for seven days.

After 7 days, the samples were removed from the incubator and the molds were split. The set CEM and MTA blocks were removed carefully by applying light force, taking care not to damage the samples. After removal, the samples were evaluated for voids or cracks. To test the compressive strength, the samples were placed lengthwise between the platens of a universal testing machine (Z050; Zwick/Roell Group, Ulm, Germany). Cross head of the device applied force at a speed of 1 mm/min in the direction parallel to the longitudinal axis of the molds until the materials were crushed. This force was recorded based on Newton’s (N) and was converted into MPa using the following formula: CS=4*p*/*μd*2 where *p* is the maximum force applied in Newton's, and *d* is the mean diameter of the specimen in mm.

Data were analyzed using the Kruskal Wallis and Dunne test. Software (SPSS version 18.0, SPSS, Chicago, IL, USA) was used for the analysis of data. The level of statistical significance was defined at 0.05.

## Results

Table 1 shows the mean (median) and standard deviation (SD) of compressive strength in eight experimental groups. In all concentrations of PG, MTA exhibited higher compressive strength compared to CEM cement. In CEM samples the results showed that adding 20% PG to CEM liquid could significantly increase the compressive strength of the samples in comparison with control group and group mixed with 100% PG.(*P*=0.047, and *P*=0.011 respectively). Group mixed with 50% PG also showed a significantly better result than 100% PG (*P*=0.028).

In MTA samples, adding 100% PG and 50% PG significantly increase the compressive strength of CEM cement in comparison with control group (*P*=0.037 and *P*=0.005, respectively). However, this difference was not significant for group mixed with 20% PG (*P*=0.084). It has also been shown that there was not a significant difference between the samples mixed with different ratio of PG (100%, 20% and 50%).

**Table 1:** Mean (SD) of compressive strength in different groups (Similar lower case and upper case letters indicate no statistically significant differences (*P*<0.05) in the same row and column, respectively

**Groups/vehicle**	**100% PG**	**20% PG**	**50% PG**	**100% CEM/MTA liquid**
**MTA**	20 (20)±4.56 ^A^a	18 (19.9)±6.31 ^A^_a__b_	22 (22.3)±2.52 ^A^_a_	10 (11)±3.20 ^A^_b_
**CEM**	0.72 (0.72)±0.15 ^B^_c_	1.83 (1.93)±0.44 ^B^_a_	1.63 (1.56)±0.41 ^B^_ab_	0.84 (0.97)±0.37 ^B^_bc_

## Discussion

Since the introduction of MTA and CEM cement, various methods or vehicles have been used to improve their characteristics [13, 28-32]. For instance, by removing gypsum at the final stage of the manufacturing process and adding polycarboxylate super plasticizers, the setting time of MTA decreased and its flowability increased [13] or by adding 10% calcium chloride to CEM cement, solubility, pH and setting time of this cement improved [32]. Other researchers studied the influence of different vehicles on physical and chemical properties of MTA [3, 6, 7, 28]. As stated before, PG was added to MTA to improve its handling. It has also been shown that this vehicle could increase its bond strength [10]. The results of the present study showed that all concentrations of PG increased the compressive strength of MTA. This increase was statistically significant for concentrations of 100% and 50%.

On the other hand, Ghasemi *et al.* [33] showed that Mixing MTA with 20% PG significantly reduced the CS. This difference may be attributed to the different experimental set ups that have been used in two studies. Ghasemi *et al. *[33] used paraffin to grease the internal surfaces of their steel molds before material placement. Paraffin and PG may have a chemical interaction which could adversely affect the compressive strength of MTA.

Salem Milani *et al. *[10] showed that mixing MTA with 100% and 20% PG increased its push-out bond strength to dentin but the most suitable ratio was 80% DW-20% PG which is partly in accordance with the present study although these two studies are not directly comparable.

Based on the results of the present study, adding PG to the CEM cement in the concentration of 20% could significantly increase the compressive strength of the samples in comparison to the control group. Adding 100% PG to this cement not only didn’t increase but also caused a non-significant decrease in the compressive strength value. As the CS of hydraulic cements is an indicator of hydration reaction, this finding may be attributed to the change in the hydration process of powder particles when CEM cement mixed with 100% PG. 

There are no published data available on the CS of CEM cement when PG was used as a vehicle; thus direct comparison with other studies is impossible.

Another finding of this study was that in all concentration of PG, MTA samples showed better results than CEM cement. This finding is not in agreement with Shahi *et al.* [17] who reported that irrespective of the differences in mixing techniques, the CS of CEM cement is similar to MTA after 21 days. This difference could be attributed to different experimental set-ups, different time intervals and different mixing methods used in these studies.

Adl *et al.* [34] reported that CEM cement showed significantly lower bond strength to the dentinal wall compared to MTA which is partly in agreement with the present study. However, as compressive strength and posh-out bond strength tests have different entities, direct comparison of the two studies is not reasonable and further studies on the effect of PG on the bond strength of CEM cement are recommended. 

## Conclusion

Under the limitations of this study, where the compressive strength is important, the use of PG in concentrations of 50% and 100% for MTA and 20% for CEM cement is cautiously recommended.
